# Efficacy and safety of high-intensity focused ultrasound ablation under general anesthesia in older hepatocellular carcinoma patients

**DOI:** 10.3389/fonc.2025.1702332

**Published:** 2025-12-11

**Authors:** Siyu Xie, Lifeng Ran, Wei Yang, Kun Zhou, Chengbing Jin, Hui Zhu

**Affiliations:** Clinical Center for Tumor Therapy, Second Affiliated Hospital of Chongqing Medical University, Chongqing, China

**Keywords:** ablation, acoustic therapy, older patients, hepatocellular carcinoma, high-intensity focused ultrasound

## Abstract

**Background:**

Older patients often have comorbidities, leading to a higher risk of delayed recovery while under general anesthesia. High-intensity focused ultrasound (HIFU) for patients with hepatocellular carcinoma (HCC) usually requires general anesthesia to alleviate procedure-related pain and ensure immobilization. This study aimed to evaluate the safety and efficacy of HIFU for the treatment of older patients with HCC, and to explore the associated prognosis and risk factors.

**Methods:**

A total of 174 patients with HCC who underwent HIFU were enrolled in this study and divided into three age groups: <60 years (n=100), 60–74 years (n=55), and ≥75 years (n=19). The risks of general anesthesia associated with HIFU were observed, and the ablation effects of HIFU were evaluated using contrast-enhanced CT or MRI. Survival analysis, prognosis and risk factors were analyzed.

**Results:**

The prevalence of comorbidities was 73.68%, 52.72%, and 26.00% in patients aged ≥75 years, 60–74 years, and <60 years, respectively. The difference between the three groups was significant (*P* < 0.001). The rates of complete response (CR), partial response (PR), and ORR were 25.61%, 46.34%, and 71.95% in patients aged <60 years, 34.04%, 40.42%, and 74.46% in patients aged 60–74 years, and 12.50%, 62.50%, and 75.00% in patients aged ≥75 years, respectively. 1-year and 2-year overall survival (OS) rates in patients aged <60, 60–74, and ≥75 years were 78.4% and 70.5%, 84.5% and 75.3%, and 72.4% and 43.4%, respectively. There were no significant differences in ORR and OS between the three groups (*P* = 0.937 and 0.055, respectively). No significant difference of the median interval time of anesthesia recovery between three groups occurred (*P* = 0.208) and no other anesthesia-related complications were observed. CNLC stage III was an independent prognostic factor (*P* = 0.049). AFP ≥20 ng/mL was an independent risk factor (*P* = 0.016), whereas the combination of other local treatment methods was a protective factor (*P* = 0.016).

**Conclusions:**

HIFU ablation under general anesthesia for older HCC patients is safe, feasible and effective.

## 1 Introduction

Primary liver cancer is one of the most common malignant tumors in the world and represents a significant public health challenge. Hepatocellular carcinoma (HCC), the most common subtype in primary liver cancer, accounts for 75%–85% (1). In recent years, the incidence of HCC has been steadily increasing among older patients (2). This trend may be closely related to the delayed progression of chronic hepatitis C virus (HCV) infection, the rising prevalence of non-alcoholic fatty liver disease, and the high risk of comorbid alcoholic liver disease in older patients (3, 4).

Surgical resection remains the preferred curative option for HCC, but more than 80% of patients with HCC are diagnosed at intermediate or advanced stages, losing the opportunity for surgical resection (5). In addition, older patients with HCC often have other chronic diseases, such as diabetes mellitus, hypertension, cardiovascular and cerebrovascular diseases, and pulmonary diseases (6). Therefore, overall health status in older patients with HCC is poor, and are unable to receive the optimal treatment regimen, such as surgical resection, which leads to the risk of incomplete and unstandardized treatment and ultimately poor prognosis (7).

Recently, non-surgical local treatment techniques have been applied in patients with HCC losing the opportunity for surgical resection, such as transcatheter arterial chemoembolization (TACE), high-intensity focused ultrasound (HIFU), radiofrequency ablation (RFA), percutaneous injection of anhydrous ethanol (PEI), radioactive particle implantation, microwave ablation, and radiation therapy (8, 9). The results of numerous studies have consistently shown a survival benefit for TACE compared to supportive care in patients with unresectable HCC (10–12). One study that randomized patients with unresectable HCC to either TACE or best supportive care found that the actuarial survival was significantly better in the TACE group (1 year, 57%; 2 years, 31%; 3 years, 26%) compared to the control group (1 year, 32%; 2 years, 11%; 3 years, 3%; P = .002) (10). Among local ablation therapies such as RFA, microwave ablation and PEI, RFA is most frequently used clinically. Several studies have demonstrated that RFA is as effective as resection for patients with early-stage HCC (e.g., tumors < 3 cm) (13–15). These results have not shown statistically significant differences in OS and disease-free-survival (DFS) between the RFA and liver resection treatment groups. The results from these studies support the use of RFA as an alternative to resection in early-stage HCC patients with small tumor (< 3 cm) and are recommended by guidelines published by scientific societies (16, 17). Although these non-surgical treatment methods are feasible for local treatment in HCC, there is still no standard treatment option in the clinic.

As a non-invasive method, HIFU can ablate target tumors by biological effects in the focal region induced by focused ultrasound energy, such as high temperature (65°C – 100°C), cavitation, and mechanical effect (18–20). Although thermal, cavitation, and mechanical effects can be induced by focused ultrasound energy, HIFU mainly ablates tumor tissue through thermal effects, which differ from a new focused ultrasound technique known as histotripsy (21). Histotripsy is a nonthermal focused ultrasound modality used to ablate tumor tissue by creating short, high-pressure ultrasound waves that generate local tissue pressure changes at a focal point, leading to cavitation and mechanical tissue liquefaction (22). Currently, HIFU is being used clinically to treat liver tumors.Compared with the ablation methods commonly used in the clinic such as RFA and microwave ablation, HIFU is a completely extracorporeal, non-invasive modality that has been shown to ablate hepatic tumors safely and effectively (23), particularly in tumors in difficult locations such as those adjacent to large vessels of the liver and gallbladder (9, 24). These features differ from RFA and microwave ablation and indicate that HIFU may be a promising alternative and non-invasive local ablation method for older patients with HCC. However, the HIFU process for patients with HCC generally requires general anesthesia to alleviate the pain related to the procedure and ensure immobilization, and temporary breath control, including temporary inspiratory or expiratory hold by an anesthesiologist to control tumor movement with the liver. Older patients often have other chronic diseases and are at increased risk of delayed recovery by general anesthesia due to the prolonged duration of the anesthetic activity and the presence of multiple organ dysfunction, decreased neurotransmitter release, decreased drug uptake and metabolism, as well as decreased renal blood flow and glomerular filtration rate (25). Therefore, the feasibility of safety risks may increase in older patients with HCC who undergo HIFU under general anesthesia. In addition, older patients often have cardiopulmonary disease, which can result in short duration of breath control or insufficient exposure of tumor exposure to the ribs due to limited movement of the liver caused by limited hyperventilation of the lung during the HIFU procedure. This can lead to a decrease in the absorption of HIFU energy by the target tumor, potentially affecting the response of the target tumor to HIFU in older HCC patients. Recently, there have been very few studies examining HIFU ablation under general anesthesia for the treatment of older HCC patients, with only a few studies looking at HIFU under non-general anesthesia (26). This study was carried out to evaluate the safety and efficacy of HIFU ablation under general anesthesia in the treatment of older patients with HCC and to further explore the associated prognosis and risk factors.

## 2 Methods

### 2.1 Patients

This study was approved by the ethics committee of the Second Affiliated Hospital of Chongqing Medical University (No. 2025-137). From January 2020 to December 2021, 174 of 248 consecutive patients with HCC who received HIFU at the Second Affiliated Hospital of Chongqing Medical University and met the inclusion criteria were enrolled in this retrospective study. Before enrollment, informed consent was provided from all patients and their family members.

The inclusion criteria for enrollment were as follows: (1) patients with a pathologically confirmed diagnosis of HCC or those who met the criteria for clinical diagnosis according to the 2024 edition of the Guidelines for the Diagnosis and Treatment of Primary Liver Cancer (1); (2) patients with liver function of Child-Pugh class A or B before HIFU treatment; (3) liver tumors that could be evaluated by enhanced CT or MRI images; (4) tumors could be visualized by diagnostic ultrasound of the HIFU system; (5) patients with a Karnofsky performance scale (KPS) score of 70% or higher. The exclusion criteria were as follows: (1) liver function of Child-Pugh class C; (2) tumors that could not be evaluated by enhanced CT or MRI images; (3) patients with active and uncontrolled infection; (4) presence of a tumor thrombus in the inferior vena cava without a protective filter to prevent pulmonary or systemic embolism.

Based on research on human function and lifespan, the World Health Organization (WHO) considers individuals aged ≥60 years and older to be older adults. Gerontological classifications commonly distinguish 60–74 years as the “young-older adults” and ≥75 years and older as the “old-older adults” (27, 28). To determine if older patients of different age groups can safely and effectively undergo HIFU treatment under general anesthesia, patients in this study were divided into three age groups for observation and analysis: under 60 years, 60–74 years, and ≥75 years and older.

### 2.2 HIFU treatment

#### 2.2.1 HIFU device

A Model-JC HIFU system (Chongqing Haifu Medical Tech Co., Ltd., Chongqing, China), which was equipped with a diagnostic ultrasound probe (MyLab 70, Esaote, Genova, Italy) in the center of the HIFU transducer for real-time monitoring of the treatment procedure, was used for HIFU treatment. Therapeutic ultrasound beams were produced by a 20-cm diameter transducer with focal length of 150 to 160 mm, operated at a frequency of 0.8 MHz. The details of the device have been described in our previous publications (29, 30).

#### 2.2.2 HIFU procedure

**Figure 1:**
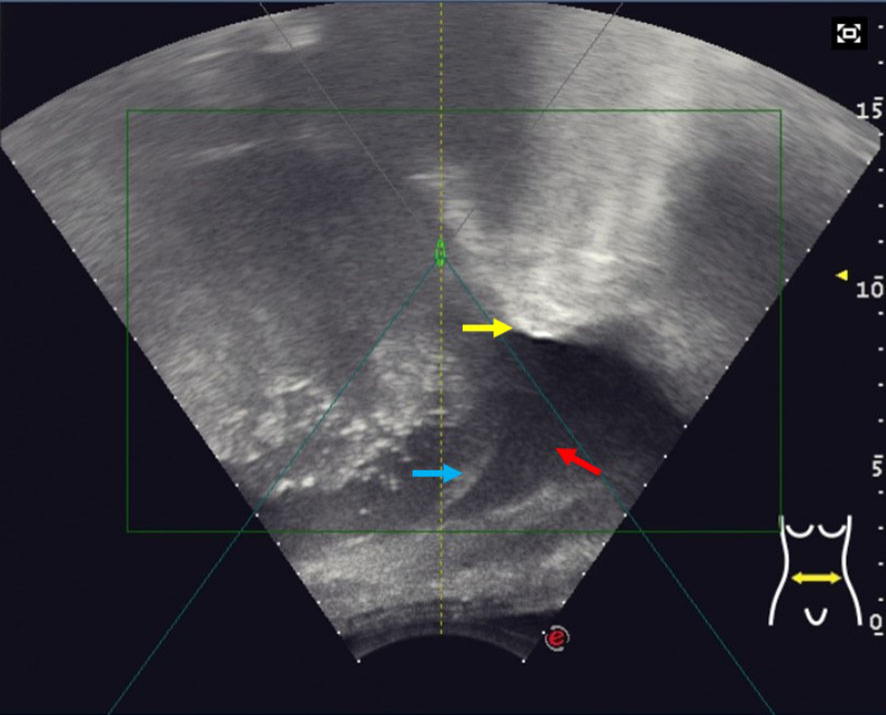
Representative ultrasound image monitoring during HIFU showing a good ultrasound pathway to remove lung tissue using the artificial pleural effusion method. The yellow arrow indicates the lung pushed by artificial pleural fluid; The blue arrow shows the diaphragma; The red arrow shows the artificial pleural fluid (injected normal saline).

**Figure 2:**
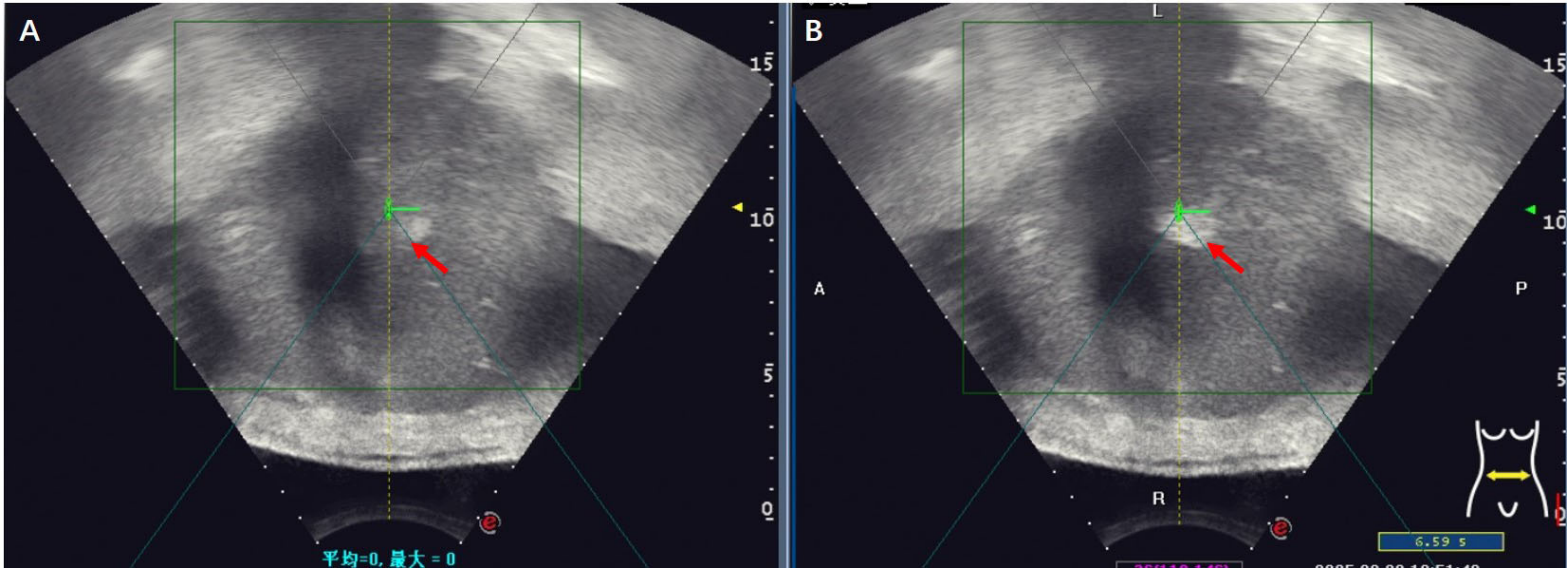
Gray-scale changes were observed on B-mode ultrasound images during HIFU procedure in a HCC patient. **(A)** Pre-HIFU: a liver lesion with ill-defined margins and heterogeneous echogenicity (red arrow); **(B)** Immediately after HIFU exposure: the grayscale is clumped and increased in the volume of the target tumor (red arrow), the grayscale change that is regarded as a sonographic marker of immediate technical success.

All patients underwent the HIFU process under general anesthesia to alleviate procedure-related pain and ensure immobilization. Enhanced CT or magnetic resonance imaging was performed prior to HIFU to determine the location of the target tumor and its relationship with adjacent normal tissue structures. These assessments facilitated the delineation of the target tumors and determined the need for artificial pleural effusion by injecting normal saline into the right thoracic cavity to push the lung and set the path of the ultrasound beam (**Figure 1**). All patients fasted before the procedure. Preoperative preparations were conducted, including skin cleaning and degassing of the treatment ultrasound beam pathway. After successful anesthesia, the patient was positioned in the HIFU treatment bed prone or on his right side according to the location of the liver tumor, so that the skin over the targeted lesion could easily be in contact with degassed water. During the HIFU procedure, the target tumor could be clearly identified on the integrated monitoring ultrasound imaging which integrated the center of the ultrasound transducer. The target tumor was defined and divided into parallel slices with 5-mm interval using real-time ultrasound monitoring imaging. A combination of linear and shot scanning track of HIFU exposure was used. The treatment process was monitored in real time using the monitoring ultrasound system, and ablation was shown to be effective when the target area was observed to show an increase in the gray scale of the clumped ultrasound or a significant increase in the gray scale compared to the baseline images (**Figure 2**). Temporary breath control including temporary inspiratory or expiratory holding by an anesthesiologist was often required during treatment to control tumor movement with the liver and exposing the tumor obscured by the ribs.

### 2.3 Follow up

All patients were followed every 3 months for two years and every 6 months thereafter. The objective response rate (ORR) was used to assess tumor response. Contrast-enhanced CT or MRI was performed 1–3 months after HIFU to evaluate the HIFU ablation effect. Tumor responses were assessed according to the modified Response Evaluation Criteria in Solid Tumors (mRECIST) (31, 32). ORR was calculated as (CR + PR)/total number of cases × 100%. Overall survival (OS) and progression free survival (PFS) were evaluated. All patients were followed up every 3 months during the first 2 years after HIFU and every 6 months thereafter until May 2024, death or loss to follow-up. Adverse events (AEs) related to HIFU occurring during HIFU treatment and within 30 days after the procedure were assessed and graded using the National Cancer Institute Common Terminology Criteria for Adverse Events (CTCAE), v.5.0 (33).

### 2.4 Statistical analysis

Statistical analysis was performed using SPSS software (v.26.0). Quantitative variables were analyzed using ANOVA or the Kruskal–Wallis (K–W) test. Categorical variables were evaluated using the chi-square test or Fisher’s exact test. The Kaplan–Meier method was used for survival analysis, and the log-rank test was used to assess survival differences between the three groups. Univariate analysis was performed using the Kaplan–Meier method and log rank test, and multifactorial Cox proportional hazards regression model analysis was used for analysis of prognostic factors. Univariate and multivariate logistic regression analyzes were used to evaluate risk factors associated with treatment efficacy. A P-value < 0.05 was considered statistically significant.

## 3 Results

### 3.1 Baseline characteristics

A total of 174 patients with HCC were divided into three groups by age (<60, 60–74, and ≥ 75 years). The baseline characteristics of the three age groups are summarized in **Table 1**. The median ages of the patients aged <60 (100 patients), 60–74 (55 patients), and ≥ 75 years (19 patients) were 51 (range: 25–59), 65 (range: 60–74), and 78 (range: 75–88), respectively. The prevalence of comorbidities was 73.68%, 52.72%, and 26.00% in patients aged ≥75 years, 60–74 years and <60 years, respectively. The difference of prevalence of comorbidities between three groups was significant (*P* < 0.001). Significant differences in the KPS score, national nosocomial infections surveillance (NNIS) classification, and anesthesia risk score (ASA) classification were observed across the three groups (*P* = 0.045, <0.001, and <0.001, respectively). No significant differences were observed in other baseline variables.

**Table 1:** Baseline characteristics of patients in the three groups.

Variable	<60 years	60–74 years	≥ 75 years	*P* value
Number of patients	100	55	19	
Age (yr)	51 (25-59)	65 (60-74)	78 (75-88)	<0.001
Gender (male/female)	82/18	47/8	16/3	0.854
Comoribidity (Yes/No)	26/74	29/26	14/5	<0.001
KPS score (≥90/80-90/70-80)	85/15/0	37/17/1	14/5/0	0.045
Child-Pugh grade (A/B)	82/18	38/17	16/3	0.141
HBsAg (+/-)	82/18	43/12	11/8	0.066
Serum bilirubin (umol/L)	12.5 (8.2,17.7)	15.4 (11.3,22.7)	10.25 (7.6,17.3)	0.069
Serum albumin (g/L)	34.6 (31.4,41.3)	34.3 (31.7,39.0)	34.3 (30.9,38.2)	0.958
NNIS classification (0/1)	81/19	32/23	7/12	<0.001
ASA classification (I-II/III)	81/19	33/22	7/12	<0.001

KPS, Karnofsky Performance Status; HBsAg, Hepatitis B Surface Antigen; NNIS, National Nosocomial Infections Surveillance; ASA, American Society of Anesthesiologists.

Most of the patients had undergone previous local therapies (TACE, surgical resection, RFA) and systemic therapy (molecular targeted therapy, immune checkpoint inhibitors). Among these, TACE was the most frequently administered treatment, received by 69.00%, 67.27%, and 63.16% of patients aged <60, 60–74, and ≥75 years, respectively. More than 80% of patients had multiple liver tumors, whereas fewer than 20% had a single tumor. No significant differences in AFP level, portal vein tumor thrombus, and China liver cancer staging (CNLC stage) were observed across the three groups. All previous treatments and tumors features are shown in **Table 2**.

**Table 2:** Baseline characteristics of tumors and previous treatment in the three groups.

Variable	<60 years (n=100)	60–74 years (n=55)	≥75 years (n=19)	*P* value
Previous local treatment				
Hepatic resection (Yes/No)	20/80	12/43	2/17	0.555
TACE (Yes/No)	69/31	37/18	12/7	0.878
RFA (Yes/No)	16/84	9/46	0/19	0.167
PEI (Yes/No)	16/84	5/50	3/16	0.473
Radioactive seed implantation (Yes/No)	7/93	0/55	0/19	0.083
Radiotherapy (Yes/No)	26/74	8/57	5/14	0.239
Previous systematic treatment				
Targeted therapy (Yes/No)	28/72	8/47	4/15	0.159
Immune checkpoint inhibitors (Yes/No)	14/86	3/52	0/19	0.072
Chemotherapy (Yes/No)	0/100	2/53	0/19	0.179
AFP (<20ng/ml/≥20ng/ml/Unmeasured)	31/58/11	24/25/6	6/9/4	0.368
Number of tumors (1/≥2)	12/88	11/44	3/16	0.407
Portal vein tumor thrombosis (vp1/vp2/vp3/vp4/No)	0/10/20/10/60	0/0/11/5/39	0/0/0/4/15	0.168
Vascular invasion (Yes/No)	19/81	10/45	4/15	0.963
Extrahepatic metastasis (Yes/No)	27/73	11/44	1/18	0.100
CNLC stage (I/II/III)	16/23/61	14/19/22	4/7/8	0.120

TACE, Transarterial Chemotherapy Embolization; RFA, Radiofrequency Ablation; PEI, Percutaneous Ethanol Injection; AFP, alpha fetoprotein; CNLC, China liver cancer staging.

### 3.2 Tumor response

The treatment-related characteristics of three groups are shown in **Table 3**. The number and size of tumors treated with HIFU were not significantly different across the three groups (*P* = 0.243 and 0.070, respectively). In all patients, the common combination therapies were TACE (35.63%) and molecular targeting therapy (35.06%), with no significant differences between three groups (*P* = 0.558 and 0.820, respectively). Energy of HIFU treatment and sonication time differed significantly between three groups (*P* = 0.003 and 0.002, respectively), and pairwise comparisons showed that it was significantly higher in patients aged <60 years than in those aged 60–74 or ≥75 years. No significant differences in the number of cases with breath control between three groups were observed.

**Table 3:** Characteristics of HIFU-treated tumors and HIFU treatment data in three groups.

Variable	<60 years (n=100)	60–74 years (n=55)	≥75 years (n=19)	*P* value
Number of treated tumors (1/≥2)	37/63	22/33	11/8	0.243
Size of treated tumors (<5cm/≥5cm)	29/71	25/30	9/10	0.070
Combined local treatment	66/34	35/20	10/9	0.539
TACE (Yse/No)	39/61	17/38	6/13	0.558
PEI (Yse/No)	24/76	15/40	2/17	0.329
Radioactive seed implantation (Yse/No)	24/76	12/43	6/13	0.692
Combined systematic treatment	46/54	23/32	7/12	0.720
Targeted therapy (Yes/No)	37/63	18/37	6/13	0.820
Immune checkpoint inhibitors (Yes/No)	19/81	8/47	1/18	0.305
Energy of HIFU (J)	289013.5 (200864.5,469300)	195621 (103183,315200)	199520 (104100,283252)	0.003
HIFU treatment power (watt)	400(369.25,400)	394(351.5,400)	383(345.5,400)	0.497
Sonication time (s)	763.5(528.5,1206)	552(318,843)	522(296,755)	0.002
Atificial pleural effusion (Yes/No)	25/75	8/47	7/12	0.105
Temporary breath control (Yes/No)	29/70	19/36	4/15	0.518
Postoperative recovery time (min)	30 (15-100)	35 (20-70)	45 (35-60)	0.208

TACE, Transarterial Chemotherapy Embolization; PEI, Percutaneous Ethanol Injection.

Contrast-enhanced CT or MRI was performed 1 to 3 months after HIFU treatment to assess short-term tumor response. Overall, 145 patients were evaluated, 29 patients (18, 8, and 3 patients in the groups of <60 years, 60–74 years, and ≥75 years, respectively) were excluded (14 failure to review images within 1–3 months after HIFU, 15 loss of follow-up).

**Figure 3:**
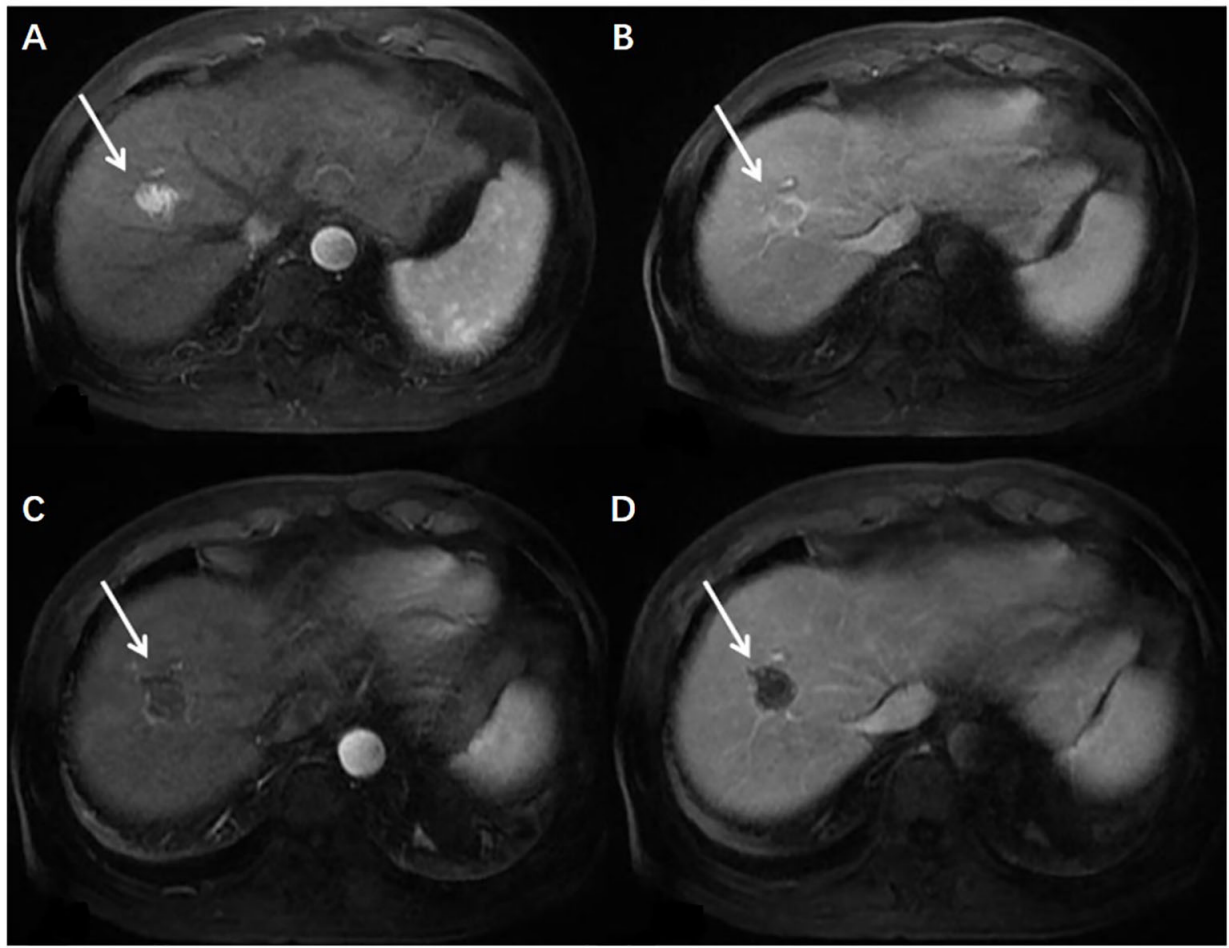
Changes in enhanced MRI images before and after HIFU in a 62-year-old male patient with HCC located in the right liver lobe (lesion size: 16 × 23 mm). **(A)** Before HIFU, the arterial phase in T1WI showed the rich blood supply in the lesion; **(B)** Before HIFU, the portal venous phase in T1WI showed partial contrast clearance in the lesion; **(C)** After HIFU, the arterial phase of T1WI did not show contrast perfusion in the lesion; **(D)** After HIFU, the portal venous phase in T1WI showed no contrast perfusion in the lesion.

According to the modified Response Evaluation Criteria for Solid Tumors (mRECIST), the disappearance of contrast agent perfusion in the target tumor on enhanced MRI or CT images after HIFU compared to before HIFU indicated effective ablation by HIFU (**Figure 3**). The complete response (CR), partial response (PR), and ORR were 25.61%, 46.34%, and 71.95% in patients aged <60 years, 34.04%, 40.42%, and 74.46% in patients aged 60–74 years, and 12.50%, 62.50%, and 75.00% in patients aged ≥75 years, respectively. No significant difference in ORR was observed across the three groups (*P* = 0.937). The details of tumor responses are shown in **Table 4**.

**Table 4:** Tumor responses after HIFU in the three groups.

Variable	<60 years (n=100)	60–74 years (n=55)	≥75 years (n=19)	*P* value
Number of patients receiving efficacy assessment(n)	82	47	16	
CR	21	16	2	/
PR	38	19	10	/
SD	15	8	3	/
PD	8	4	1	/
ORR(n(%))	59(71.95%)	35(74.46%)	12(75.00%)	0.937

CR, Complete Response; PR, Partial Response; SD, Stable Disease; PD, Progressive Disease; ORR, ObjectiveResponse Rate.

### 3.3 Survival outcomes

**Figure 4:**
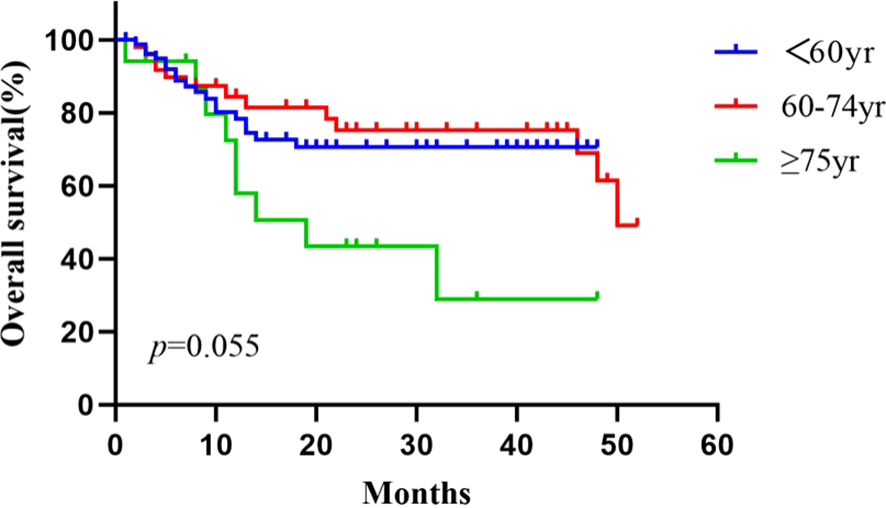
Cumulative survival curves for the <60 age, the 60–74 age, and the ≥75 age groups, using the Kaplan–Meier method. The survival time was calculated from the start of the HIFU treatment. The log rank test did not show statistically significant differences between the three groups (*P* = 0.055).

The average follow-up time for the patients aged <60 years, 60–74 years, and ≥75-years was 14.9 ± 15.5 months (range: 1–48), 22.3 ± 18.9 months (range: 2–52), and 15.2 ± 13.4 months (range: 1–48), respectively. The 1- and 2-year overall survival rates in patients aged <60, 60–74, and ≥75 years were 78.4% and 70.5%, 84.5% and 75.3%, and 72.4% and 43.4%, respectively. The median overall survival time was not reached in patients aged <60 years, and were 50.0 months and 19.0 months for those aged 60–74 and ≥75 years, respectively. During the follow up period, 40 of 174 patients died (18, 13, and 9 patients in the groups of <60, 60–74, and ≥75-years, respectively) mostly due to tumor progression (11 cases) and severe infections (9 cases). The Kaplan–Meier curves of the three groups were shown in **Figure 4**. The median PFS was 4, 7, and 4 months in patients aged <60, 60–74, and ≥75 years, respectively. No significant differences in OS or median PFS across the three groups was found (*P* = 0.055 and 0.261, respectively).

### 3.4 Adverse events related to HIFU treatment

The AEs related to treatment with HIFU are shown in **Table 5**. AEs were observed in 94 patients (54.0%). The common AEs included HIFU treatment-related pain (31.6%), elevated ALT/AST levels (54.0%), and edema of skin and soft tissue at the HIFU-irradiated area (13 patients, 7.5%). The difference of incidence of edema of skin and soft tissue at HIFU irradiation region was significant between three groups (*P* = 0.048), but subsequent pairwise comparisons showed no significant differences between any two groups. The incidence of infection differed significantly between three age groups (*P* = 0.022), with pairwise analysis indicating a significantly higher incidence in patients aged <60 years compared with those aged 60–74 years, but no significant difference was observed between the ≥75 years group and the other two age groups.

**Table 5:** Adverse events related to HIFU in the three groups.

Variable	<60 years (n=100)				60–74 years (n=55)				≥75 years (n=19)				*P* value
	G 1	G 2	G 3	G 4	G 1	G 2	G 3	G 4	G 1	G 2	G 3	G 4	
Pain	36	5	1	0	0	5	0	0	8	0	0	0	0.980
ALT	11	5	2	0	9	1	1	0	2	0	0	0	0.647
AST	17	7	7	1	17	6	1	0	5	2	0	0	0.353
Serum bilirubin	3	2	0	0	1	0	0	0	0	0	1	0	0.521
Infection	0	0	10	0	0	0	0	0	0	0	0	0	0.022
Soft tissue edema	4	0	1	0	7	1	0	0	0	0	0	0	0.048
Ascites	0	3	0	0	0	2	0	0	0	0	0	0	>.999
Hydrothorax	0	3	0	0	0	0	0	0	0	0	0	0	0.685
Abdominal distension	2	1	0	0	1	1	0	0	0	0	0	0	>.999
Diarrhea	0	0	0	0	0	1	0	0	0	0	0	0	0.425

ALT, Alamine Aminotransferase; AST, Aspartate Aminotransferase.G, grade.

One patient died after treatment due to renal failure not associated with HIFU, which was attributed to her pre-existing chronic renal insufficiency. Another patient aged <60 years of age presented a firm dark-colored cutaneous nodule at the HIFU treatment site after HIFU, which improved after debridement and suturing. AEs in other patients resolved or improved after routine symptomatic treatment. The median interval time between leaving the operating room and cessation of anesthesia in groups of <60 years, 60–74 years and ≥75-years was 30 min (range: 15–100), 35 min (range: 20–70) and 45 min (range: 35–60), respectively. No significant differences between the three groups were observed (*P* = 0.208). No delayed recovery from anesthesia or other complications related to anesthesia were observed.

### 3.5 Analysis of influencing factors related to prognosis and efficacy

The univariate analysis of prognostic factors performed using the Kaplan–Meier method and the log rank test showed the liver function of the Child–Pugh classification, the presence of comorbidities, the NNIS classification, the ASA classification, the diameter tumor size of HIFU-treated lesions, the stage of CNLC, and the combination of systemic therapy were significantly related to overall survival (*P* < 0.05). Analysis of the Cox proportional hazards regression model showed that stage III CNLC (HR = 3.695, *P* = 0.049) was an independent risk factor that affected survival. The details are shown in **Tables 6**, **7**.

**Table 6:** Univariate analysis for factors affecting the patients’ prognosis.

Variable	Numbers	1-year survival rate (%)	2-year survival rate (%)	χ^2^	*P* value
Age				5.797	0.055
<60years	100	78.4	70.5		
60-74years	55	84.5	75.3		
≥75years	19	72.4	43.4		
Gender				0.714	0.398
Male	145	76.6	68.2		
Female	29	86.2	67.1		
KPS score				5.857	0.053
90	136	82.1	71.7		
80	37	61.8	56.2		
70	1	-	-		
Child-Pugh grade				6.094	0.014
A	136	82.7	72.8		
B	38	55.3	47.4		
HBsAg				1.732	0.188
+	136	76.4	73.4		
-	38	82.6	54.2		
AFP				4.310	0.116
<20ng/ml	61	89.3	78.4		
≥20ng/ml	92	72.2	65.7		
Unmeasured	21	65.3	49.0		
Comorbidity				4.188	0.041
Yes	69	68.9	64.1		
No	105	87.7	71.3		
NNIS classification				7.554	0.006
0	120	84.8	75.9		
1	54	60.9	50.3		
ASA classification				6.518	0.011
I-II	121	87.7	74.5		
III	53	59.7	52.7		
Number of HIFU-treated tumors				2.873	0.090
1	70	85.6	79.2		
≥2	104	76.0	59.5		
Size of HIFU-treated tumors				13.702	<0.001
<5cm	62	93.5	88.3		
≥5cm	112	68.8	56.3		
CNLC stage				36.219	<0.001
I	34	100.0	91.0		
II	49	92.0	88.6		
III	91	57.2	42.3		
Combined local treatment				1.384	0.239
Yes	111	74.3	64.5		
No	63	84.8	75.6		
Combined systematic treatment				19.380	<0.001
Yes	76	63.2	52.3		
No	98	88.3	79.6		

KPS, Karnofsky Performance Status; HBsAg, Hepatitis B Surface Antigen; AFP, alpha fetoprotein; NNIS, National Nosocomial Infections Surveillance; ASA, American Society of Anesthesiologists; CNLC, China Liver CancerStaging.

**Table 7:** Multifactorial Cox proportional hazards regression model analysis for prognosis factors.

Variable	β	SE	Wald	*P* value	HR (95%CI)
Child-Pugh grade (A/B)	0.491	0.375	1.711	0.191	1.634 (0.783-3.411)
Comoribidity (Yes/No)	-0.018	0.340	0.003	0.957	0.982 (0.505-1.911)
ASA classification (I-II/III)	0.604	0.353	2.926	0.087	1.830 (0.916-3.656)
Size of treated tumors (<5cm/≥5cm)	0.748	0.487	2.358	0.125	2.114 (0.813-5.494)
CNLC stage					
I					Reference
II	-0.555	0.787	0.498	0.481	0.574 (0.123-2.685)
III	1.307	0.664	3.873	0.049	3.695 (1.005-13.583)
Combined systematic treatment (Yes/No)	0.691	0.377	3.359	0.067	1.996 (0.953-4.179)

ASA, American Society of Anesthesiologists; CNLC, China Liver CancerStaging.

Logistic regression analysis was used to evaluate the risk factors associated with treatment efficacy. Univariate analysis showed that the AFP level, NNIS classification, ASA classification, number of tumors treated, CNLC stage, and combination of other local treatment methods were related to treatment efficacy (*P* < 0.1). Multifactorial analysis showed that AFP ≥20 ng/mL was an independent risk factor for poor efficacy (OR = 3.804, *P* = 0.016), whereas the combination of other local treatment methods was a protective factor (OR = 0.335, *P* = 0.016). The details are shown in **Table 8**.

**Table 8:** Univariate and multivariate logistic analysis for factors affecting efficacy in patients.

Variable	Univariate analysis			Multivariate analysis		
	OR	95%CI	*P* value	OR	95%CI	*P* value
Age						
<60years	Reference					
60-74years	0.880	0.390-1.984	0.757			
≥75years	0.855	0.250-2.925	0.803			
Gender (Male/Female)	1.051	0.382-2.893	0.924			
KPS score (90/80/70)	1.911	0.828-4.410	0.129			
Child-Pugh grade (A/B)	1.214	0.501-2.942	0.667			
HBsAg (+/-)	1.259	0.515-3.080	0.613			
AFP (<20ng/ml/≥20ng/ml)	3.067	1.149-8.188	0.025	3.804	1.285-11.258	0.016
Comoribidity (Yes/No)	1.089	0.519-2.286	0.822			
NNIS classification (0/1)	2.035	0.939-4.411	0.072			
ASA classification (I-II/III)	2.140	0.985-4.653	0.055	2.256	0.927-5.489	0.073
Number of HIFU-treated tumors (1/≥2)	2.188	0.988-4.846	0.054	1.774	0.716-4.396	0.216
Size of HIFU-treated tumors (<5cm/≥5cm)	1.310	0.597-2.875	0.501			
CNLC stage						
I	Reference			Reference		
II	4.333	1.108-16.951	0.035	2.369	0.529-10.615	0.260
III	3.981	1.096-14.459	0.036	2.789	0.691-11.255	0.150
Combined local treatment (Yes/No)	0.505	0.237-1.072	0.075	0.335	0.138-0.813	0.016
Combined systematic treatmen (Yes/No)	1.359	0.648-2.851	0.417			

KPS, Karnofsky Performance Status; HBsAg, Hepatitis B Surface Antigen; AFP, alpha fetoprotein; NNIS, National Nosocomial Infections Surveillance; ASA, American Society of Anesthesiologists; CNLC, China Liver Cancer Staging.

## 4 Discussion

Surgical resection remains the preferred curative option for HCC (5). However, older patients with HCC usually do not receive the optimal treatment such as surgical resection, because these patients usually have a poor overall health status due to the presence of other chronic diseases (6, 7). Recently, non-surgical local treatment techniques have been applied to patients with HCC who lose the opportunity for surgical resection, such as TACE, RFA, cryoablation, and laser ablation (8, 9). Although these non-surgical local treatment methods are feasible for local treatment in older patients with HCC, no standard treatment option has been defined in the clinic. Compared with the ablation methods commonly used in the clinic such as RFA and microwave ablation, HIFU is a completely extracorporeal, non-invasive modality that has been shown to ablate hepatic tumors safely and effectively (23), particularly in tumors in difficult locations such as those adjacent to large vessels of the liver and gallbladder (9, 24). In addition, a large liver tumor (diameter of ≥5 cm) in patients with HCC can be ablated by HIFU safely and effectively (34). These features differing from RFA and microwave ablation indicate that HIFU may be a promising alternative and non-invasive local ablation method for older patients with HCC.

The prevalence of comorbidities and indicators of general health status (KPS score, NNIS classification, and ASA classification) were significantly higher in the 60–74-year-old and ≥75-year-old groups compared with the <60-year-old group in our study, indicating that older patients had poorer general conditions and a higher incidence of comorbidities (35). Older patients often present comorbidities and are at increased risk of delayed recovery during general anesthesia. This is often caused by the prolonged duration of anesthetic drug action due to the presence of multiple organ dysfunction, reduced neurotransmitter release, decreased drug uptake and metabolism, as well as decreased renal blood flow, and glomerular filtration rate (25). Furthermore, the use of temporary breath holding to control the movement of the tumor with the liver during HIFU can result in carbon dioxide retention or inadequate oxygenation, possibly affecting brain metabolism and further delaying postoperative recovery from anesthesia. Although all patients in this study received HIFU under general anesthesia and breath holding was temporary in some patients, the median interval time between leaving the operating room and cessation of anesthesia was not significantly different across the three groups and no delayed anesthesia recovery or other anesthesia-related complications were observed. These results suggest that general anesthesia and temporary breath control are safe approaches in older patients with HCC in the treatment of HIFU, similar to results in younger patients. However, these results contradict our initial hypothesis that older patients have a higher risk of anesthesia. This discrepancy may be related to the fact that HIFU is a completely non-invasive treatment with minimal trauma and no risk of bleeding, as well as the short duration of the procedure and the reduced need for anesthetic agents (36). Further studies are needed to confirm these results and reasons.

In this study, ORR in the <60 years, 60–74 years, and ≥75 years age groups were 71.95%, 74.46%, and 75.00%, respectively. No significant difference between three groups were observed, indicating that older patients with HCC can receive the similar efficacy. However, the short-term ablation effect of target tumors produced by HIFU should be evaluated within 3 months after the procedure. In this study, 29 patients were excluded from the analysis: 18 were under 60 years old, 8 were between 60–74 years old, and 3 were 75 years or older. Out of these 29 cases, 14 did not undergo enhanced CT or MRI within 3 m after HIFU, and 15 were lost to follow-up, leading to an evaluation failure. It is important to note that this may introduce bias into the results. The 1-year and 2-year overall survival rates in patients aged <60, 60–74, and ≥75 years were 78.4% and 70.5%, 84.5% and 75.3%, and 72.4% and 43.4%, respectively. In addition, the median PFS was 4, 7, and 4 months in patients aged <60, 60–74, and ≥75 years, respectively. No significant differences in OS or in median PFS between three groups were identified. These results suggest that age has no significant influence on survival in patients with HCC treated with HIFU.

Although previous studies have addressed the prognostic factors of HIFU in the treatment of HCC, they remain undefined. Cheung et al. reported that HIFU for small-sized HCC lesions may confer a greater survival benefit (37). However, no significant correlation between lesion diameter and clinical outcomes has been reported, whereas the ECOG performance status score of 1 and multiple tumors have been identified as independent prognostic factors in patients treated with HIFU (26). In this study, stage III CNLC was an independent risk factor that affected survival in patients with HCC treated with HIFU.

Tumor size and elevated AFP levels have previously been identified as risk factors for incomplete tumor ablation after HIFU treatment (38). This study did not show correlation between tumor size and tumor response, whereas AFP ≥ 20 ng/mL was an independent risk factor. The elevated level of AFP in patients with HCC is generally related to more aggressive tumor biology and a higher tumor burden, indicating poor treatment efficacy (38). Furthermore, the combination of other local treatment methods was a protective factor for the efficacy of HIFU treatment. This suggests that combination therapy may synergistically improve HIFU efficacy through multiple mechanisms such as increased heat sink and changes in ultrasound environment, similar to previous studies (39, 40). Therefore, a combination of appropriate local treatment modalities can improve efficacy.

Skin burns and pain in the HIFU-treated area are the main AEs observed in patients with HCC treated with HIFU (41). In our study, one patient aged <60 years presented a firm, dark-colored skin nodule at the HIFU treatment site after HIFU, which improved following debridement and suturing. Previous studies have shown that sonication time, total energy deposited, distance from the focal region to the skin, abdominal wall scar and abdominal wall thickness are significantly correlated with skin burns (42, 43). Enhanced MRI images prior to HIFU treatment revealed that the lesion in this patient was located beneath the liver peritoneum near the surface of the abdominal wall. The distance from the focal region (targeted tumor) to the skin was short (2.0 cm) and the total energy was 63,504 J (sonication time 378 s, mean acoustic power 168 W). Skin burns are most likely result of the short distance between the skin and the targeted tumor, which increases the deposition of near-field energy from the focal volume along the skin on the ultrasound pathway. Consistent with previous studies, such cases require careful selection of the ultrasound beam path, lower acoustic power, and longer inter-sonication intervals to reduce the risk of skin injury (30, 42, 43). The main AEs in this study included increased liver enzyme levels and pain in the treated area, all of which improved with symptomatic treatment. The incidence of infection after HIFU was significantly different between three groups, with pairwise comparisons showing a significant difference between patients aged <60 years and those 60–74 years old. The reasons may be the proportion of tumor size treated with HIFU in diameter of ≥5 cm and the number of ≥2 tumors were higher in the <60-year group than in the other two groups, leading to a larger therapeutic target volume and a greater number of target tumors should be ablated and produce a necrosis of a larger tumor volume. These larger volumes of necrotic tissues in the body may facilitate bacterial reproduction and increase the risk of infection.

This study found that the total energy and sonication time of HIFU treatment were significantly higher in patients aged <60 years than in those aged 60–74 and ≥75 years. This difference may be related to several factors. The percentage of patients treated with HIFU with a total tumor size ≥5 cm in diameter was 71.0% in the <60-year-old group, which was higher than that in the 60–74-year-old group (54.5%) and in the ≥75-year-oldgroup (52.6%).Additionally, the proportion of patients with HIFU-treated lesions with a tumor number of ≥2 in the <60-year-old group was 63.0%, higher than that in the 60–74-year-old group (60.0%) and in the ≥75-year-old group (42.1%). These results suggest that higher HIFU energy and longer sonication time for patients aged <60 years should be required to ablate a larger target tumor volume and more lesions in order to achieve effective ablation (34, 44).However, the energy of HIFU irradiation for patients is also related to other factors, including target tumor tissue properties, heat sink effects in the target volume, and energy attenuation influenced by the ultrasound beam pathway. Future studies should be performed to clarify their influence on HIFU energy parameters. To further evaluate the relationship between energy parameters and complications, we analyzed the total energy, HIFU treatment power, and sonication time in patients who did and did not develop postoperative infections. Our findings revealed no significant variances in these three factors between the infection and non-infection cohorts (*P* = 0.117, 0.183, and 0.127, respectively).

Although older patients with HCC often have comorbidities that may increase the risk of safety or impact the efficacy of HIFU treatment, the results of this study found that all patients received HIFU treatment safely and successfully, and there were no significant differences in ablation efficacy or survival across the three age groups, indicating that HIFU treatment of older patients with HCC is safe and effective. However, our study has some limitations that should be acknowledged. First, this was a retrospective study from a single center, which can lead to biased results. In addition, scales such as the Geriatric-8 (G-8) and the Vulnerable Elders Survey-13 (VES-13) that more comprehensively and accurately assess the health status of patients were not used. In short, multicenter and prospective studies are warranted to confirm our findings.

## 5 Conclusions

HIFU is a safe and effective treatment modality for patients with HCC, and no significant differences were found in survival outcomes and ablation effects in older patients compared to younger patients. Advanced age should not be considered a limiting factor of HIFU in the treatment of patients with HCC. In order to achieve successful HIFU ablation in patients with HCC, general anesthesia is often necessary to minimize pain, ensure immobilization, and assist with temporary respiratory control. However, this can increase the risk of general anesthesia in older patients, potentially leading to delayed recovery. Despite this, our study discovered that general anesthesia is a safe option for older HCC patients undergoing HIFU treatment, with similar outcomes to younger patients. This suggests that HIFU under general anesthesia is a viable option for older HCC patients. However, large randomized controlled clinical trials are needed to further clarify the safety and efficacy of HIFU in the treatment of older patients with HCC.

## Data Availability

The raw data supporting the conclusions of this article will be made available by the authors, without undue reservation.
